# Characterization of Activity of a Potential Food-Grade Leucine Aminopeptidase from Kiwifruit

**DOI:** 10.4061/2010/517283

**Published:** 2010-11-04

**Authors:** A. A. A. Premarathne, David W. M. Leung

**Affiliations:** School of Biological Sciences, University of Canterbury, Private Bag 4800, Christchurch 8140, New Zealand

## Abstract

Aminopeptidase (AP) activity in ripe but firm fruit of *Actinidia deliciosa* was characterized using L-leucine-p-nitroanilide as a substrate. The enzyme activity was the highest under alkaline conditions and was thermolabile. EDTA, 1,10-phenanthroline, iodoacetamide, and Zn^2+^ had inhibitory effect while a low concentration of dithiothreitol (DTT) had stimulatory effect on kiwifruit AP activity. However, DTT was not essential for the enzyme activity. The results obtained indicated that the kiwifruit AP was a thiol-dependent metalloprotease. Its activity was the highest in the seeds, followed by the core and pericarp tissues of the fruit. The elution profile of the AP activity from a DEAE-cellulose column suggested that there were at least two AP isozymes in kiwifruit: one unadsorbed and one adsorbed fractions. It is concluded that useful food-grade aminopeptidases from kiwifruit could be revealed using more specific substrates.

## 1. Introduction

Kiwifruit (*Actinidia *spp.) is an important commercial crop in New Zealand. The fruit contains a high level of a cysteine endopeptidase called actinidin (E.C. 3.4.22.14) found in the cortex of the fruit [1]. Due to this proteolytic activity of kiwifruit, it has been used to tenderize meat and prevent gelatin-based jelly from setting.

Aminopeptidases (APs), particularly those from microbial sources, are important food processing enzymes and are widely used to modify proteins in food [2–4]. Animal waste products were also investigated as a potential source of useful APs [5]. It is also possible that APs from plants could be of use in the food processing industry [6]. Recently, it has been demonstrated that APs of cabbage leaves or chickpea cotyledons can be used to catalyze the hydrolysis of peptide bonds including those of hydrophobic bitter peptides in soy protein hydrolysates, resulting in the less bitter or bland taste products which have food processing applications [7, 8]. However, for debittering protein hydrolysates or other food processing needs an attractive alternative would be to use APs from fruits grown commercially that are normally consumed fresh such as kiwifruit, which have the merit of already being generally regarded safe for the food processing industry. 

Generally, there are many studies on seed aminopeptidases [9–11] but there is a paucity of information on the occurrence and characteristics of AP activities in fruits. Importantly, since there is no prior study on AP from kiwifruit, a prerequisite towards the goal of evaluating use of APs from this fruit for food processing applications is an investigation into the occurrence and biochemical characteristics of aminopeptidase (AP) activity of kiwifruit. Here, using L-leucine-*p*-nitroanilide (L-leu-*p*-NA) as a substrate, localization and some basic biochemical characteristics of AP activity within the fruit of *Actinidia deliciosa*, and an attempt to partially purify the enzyme that is normally sufficient for food-grade enzymes are reported here.

## 2. Materials and Methods

### 2.1. Enzyme Extraction

Ripe but firm kiwifruit (*Actinidia deliciosa* cv. Hayward) was obtained from a local supermarket in Christchurch, New Zealand. Unless indicated otherwise, the whole kiwifruit was peeled and cut into small pieces before enzyme extraction. Kiwifruit tissues were ground in a mortar and pestle while adding 0.1 M of potassium phosphate buffer pH 8.0 supplemented with 1% (w/v) insoluble polyvinyl polypyrrolidone (PVPP), 5% (v/v) glycerol and 3 mM DTT. The ratio of weight of tissue (g) to volume of extraction buffer (ml) was 2 : 1. The homogenate was filtered through 2 layers of synthetic cloth and centrifuged at 10,000 × g for 20 min at 4°C. The supernatant was carefully removed and used as crude extract of the whole fruit. The extraction process was carried out in a cold room or on an ice bath.

### 2.2. Determination of Total Protein Concentration

The protein concentration in extracts was determined based on the Coomassie brilliant blue dye-protein binding principle [12]. A protein standard curve was prepared using serial dilutions of BSA (bovine serum albumin; BDH, England).

### 2.3. Determination of Aminopeptidase (AP) Activity

Aminopeptidase activity was determined as described below unless indicated otherwise using L-leucine-*p*-nitroanilide (L-Leu-*p*-NA) as a substrate. The substrate solution was prepared by dissolving 20 mg of L-Leu-*p*-NA (Sigma, St. Louis, USA) in one ml of dimethyl sulfoxide (Sigma, St. Louis, USA) and adjusting the volume to 20 ml with 0.01 M potassium phosphate buffer (pH 8.0). It was found to be stored better at −20°C for use later if prepared at pH 8.0 than at higher pH. The reaction mixture contained 0.45 ml of 0.1 M potassium phosphate buffer at pH 8.0, 0.45 ml substrate solution, and 150 *μ*l enzyme extract in Eppendorf tubes kept on ice. The control tube contained the same reaction mixture except that the enzyme extract had previously been boiled for 5 min in a water bath at 100°C and centrifuged afterwards. All the tubes were vortexed, and incubated for 1 h in a water bath at 37°C. After the incubation period, they were placed in a water bath at 100°C for 5 min to stop the enzyme reaction. After this, 0.45 ml distilled water was added to all the tubes, vortexed. and then centrifuged for 10 min at 10,000 × g at room temperature. The supernatants were carefully transferred to the cuvettes and the absorbance was measured at 410 nm. One unit of enzyme activity is defined as a change in one unit of absorbance per h at 37°C.

### 2.4. Effect of Temperature on AP Activity

The effect of temperature on AP activity was determined in three different experiments. To find the optimum temperature for the enzyme activity, AP activity in crude extracts of the whole fruit was determined at different incubation temperatures ranging from 25°C to 70°C for 1 h. In another experiment to investigate thermal stability, 150 *μ*l of the enzyme extracts were pre-incubated with 0.45 ml of potassium phosphate buffer (pH 8.0) for 30 min at the above testing temperatures. After preincubation, the substrate was added to initiate the enzyme reaction for AP activity determination at 37°C for 1 h.

### 2.5. Effect of pH on AP Activity

The effect of pH on AP activity in the crude extracts of fruit was determined by replacing the potassium phosphate buffer at pH 8.0 in the assay mixture, with the three buffer mixtures (25.0 mM acetic acid, 25.0 mM MES, and 50.0 mM Tris) at different pH values ranging from 6 to 10 as described in [13]. Then AP activity was determined.

### 2.6. Effect of Different Classes of Proteolytic Enzyme Inhibitors and Promoters on AP Activity

Crude enzyme extracts were preincubated with 0.45 ml of 0.1 M potassium phosphate buffer (pH 8) in the presence of different inhibitors or activators for 30 min at 37°C. After pre-incubation, the enzyme reaction was initiated by the addition of the substrate solution (L-leu-*p*-NA) and AP activity was determined. Concentration of activators in the reaction mixture during pre-incubation was 1.0 or 10.0 mM. The chemicals tested were EDTA, 1, 10-phenanthroline, PMSF, DTT, iodoacetamide, and NEM.

### 2.7. Effect of Divalent Cations on AP Activity

The crude enzyme extracts were pre-incubated at 37°C for 30 min with 0.45 ml of 0.1 M potassium phosphate buffer in the presence of the chlorides of Mn^2+^, Co^2+^, Ni^2+^, Mg^2+^, Ca^2+^, or Zn^2+^. The concentration of divalent cations in the reaction mixture during pre-incubation was 1.0 or 10.0 mM. After pre-incubation, the substrate solution (L-leu-*p*-NA) was added to start the enzyme reaction and AP activity was determined.

### 2.8. Partial Purification of Aminopeptidase

The whole kiwifruit (550 g) was cut into small pieces and homogenized in 225 ml of 0.1 M of potassium phosphate buffer (pH 8.0) supplemented with 1% (w/v) insoluble PVPP, 5% (v/v) glycerol, and 3 mM DTT (extraction buffer). The homogenate was filtered through 2 layers of synthetic cloth. The filtrate was centrifuged at 10,000 × g at 4°C for 20 min, and the supernatant was removed and used as crude extract. Solid ammonium sulphate ((NH_4_)_2_SO_4_) was added to the crude extract, and the resulting 25–75% precipitate was dissolved in 7.5 ml of 0.01 M potassium phosphate buffer containing 10% (v/v) glycerol and 0.2 mM DTT (buffer A). After dialysis of the 25–70% ammonium sulphate fraction against buffer A, a DEAE cellulose column (10 × 2 cm) was used to separate the fractions. Unbound proteins were eluted with buffer A, and then bound proteins were eluted with 100 ml of the buffer A containing a linear gradient of 0.00–1.0 M KCl.

### 2.9. Statistical Analysis

Statistical analysis of the data was performed using STATISTIX 8.0 software. The comparison between treatments was analysed using one-way analysis of variance (ANOVA). Where a statistical significance was observed, a Tukey's Honest Significance Difference (HSD) test was performed to determine how significant from the appropriate zero the values were. Standard errors were calculated and graphically represented as symmetrical error bars.

## 3. Results and Discussion

### 3.1. Aminopeptidase (AP) Activity in Different Parts of Actinidia deliciosa Fruit

In preliminary experiments, when crude extracts from the whole fruit had been prepared with sodium phosphate or potassium phosphate buffer (pH 7.0), AP activity was not detectable. Kiwifruit contains more than 80 volatile aroma, and flavour compounds including terpenses, esters, aldehydes, alcohols with varying levels of monoterpenes, and phenolic compounds [14, 15]. These compounds could have interfered with aminopeptidase isolation and activity. Here, a reliable protocol (as described in Section 2) for extraction of AP from kiwifruit and determination of its activity using L-leucine-*p*-nitroanilide (L-leu-*p*-NA) as a substrate has been established. The present study has established for the first time that kiwifruit has AP activity and some useful parameters with respect to its extraction, assay, stability, localization and purification.

AP activity was found in all parts of the fruit of *A. deliciosa* at different levels. The highest specific (units/mg soluble protein) and total (units/g fresh weight) AP activity was localized in the seed followed by the core, inner and outer pericarp, respectively, (Table 1). In contrast, higher enzyme activities were found in the hypodermis of fully ripe grape berries than in the seed or flesh [13].

### 3.2. Effects of pH and Temperature on Kiwifruit AP Activity

**Figure 1:**
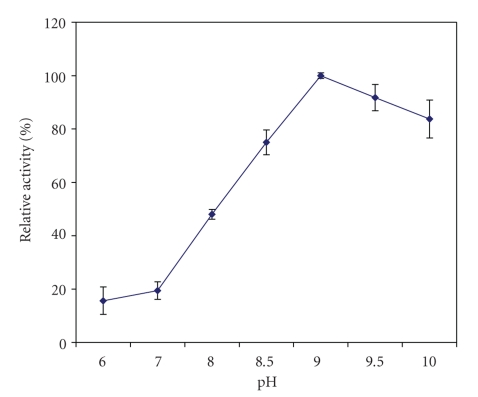
Effect of pH on aminopeptidase activity in extracts of the whole fruit of *A. deliciosa*. The enzyme activity at pH 9 was taken as 100%. Mean values of three different extracts ± standard errors are presented.

AP activity in crude extracts of the whole kiwifruit was most active at alkaline pH (Figure 1; ANOVA, *P* < .05). Similarly, hydrolysis of L-leu-*p*-NA by crude extracts was most active at a range of alkaline pH values from many different plants including potato [16], *Arabidopsis thaliana *[17], tomato [18], and daylily flowers [19].

**Figure 2:**
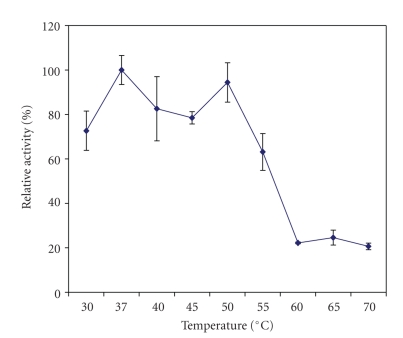
Effect of temperature on the aminopeptidase activity in the crude extracts prepared from the whole fruit of *A. deliciosa*. The enzyme activity at 37°C was taken as 100%. Mean values of three different extracts ± standard errors are presented.

**Figure 3:**
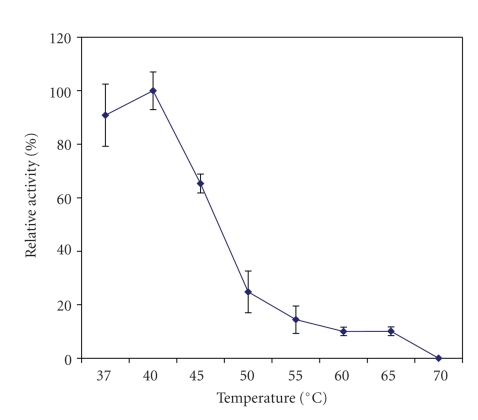
Effect of temperature on the stability of aminopeptidase activity in crude extracts prepared from the whole fruit of *A. deliciosa*. The enzyme activity at 40°C was taken as 100%. Mean values of three different extracts ± standard errors are presented.

Kiwifruit AP was most active at 37°C and 50°C, suggesting the presence of two aminopeptidase isozymes. At 55°C its activity was reduced to 63% (with the activity at 37°C designated as 100%) and then to about 20% at 60–70*º*C (Figure 2). It was most stable at 37–40°C (Figure 3) but became unstable as only less than 15% of its activity remained at temperatures higher than 55°C (ANOVA, *P* < .05).

### 3.3. Effects of Protease Inhibitors, Activators, and Metal Ions

**Figure 4:**
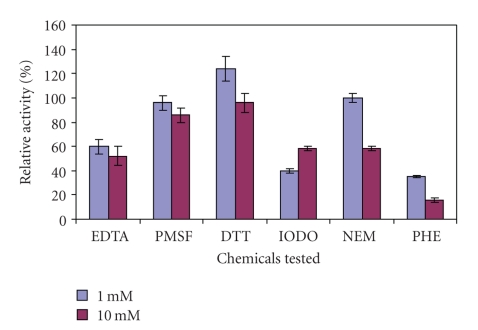
Effect of proteolytic enzyme inhibitors and activators on aminopeptidase activity in crude extract of the whole fruit of *A. deliciosa*. Enzyme activity in the absence of any chemical (control) was taken as 100%. Mean values of three different extracts ± standard errors are presented.

The presence of 1 mM of 1,10-phenanthroline, EDTA-Na_2_  and iodoacetamide inhibited kiwifruit aminopeptidase activity (Figure 4). In contrast, 1 mM of DTT had a slight stimulatory effect (ANOVA, *P* < .05). The same concentration (1 mM) of NEM and PMSF had no effect. At 10 mM, 1,10-phenanthroline, NEM, iodoacetamide, and EDTA-Na_2_ caused more inhibition. But 10 mM of DTT and PMSF had neither stimulatory nor inhibitory effect (ANOVA, *P* < .05).

The observed inhibition of kiwifruit AP activity by metal chelators such as 1,10-phenanthroline and EDTA suggested the involvement of a metal ion in the active site of the enzyme. Similar effects were also reported in the studies on leucine aminopeptidases of potato [16], tomato, *E. coli* pep A, and porcine LAPs [18]. Furthermore, DTT (a thiol reducing agent) at a lower concentration (1 mM) had a stimulatory effect but an inhibitory effect at a higher concentration on kiwifruit AP activity suggesting that it was a thiol-dependent metalloprotease rather than a cysteine protease [20]. On the other hand, iodoacetamide (1 mM) and NEM (10 mM), the specific inhibitors of cysteine protease, had 60% and 40% inhibition of kiwifruit AP activity, respectively, suggesting that cysteine residues were likely involved in the enzyme conformation rather than catalysis. A serine-type protease might not be a significant contributor to the kiwifruit AP activity as PMSF, a serine protease inhibitor, did not have any significant effect on its activity. 

**Figure 5:**
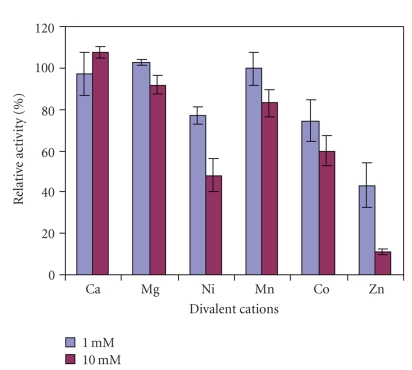
Effect of divalent cations on aminopeptidase activity in crude extracts of the whole fruit of *A. deliciosa*. Enzyme activity in the absence of any cations (control) was taken as 100%. Mean values of three different extracts ± standard errors are presented.

The effects on kiwifruit AP activity of Ca^2+^, Mg^2+^, Co^2+^, Ni^2+^, Mn^2+^, and Zn^2+^ with chloride as the counter ion were studied (Figure 5). At metal ion concentrations of 1 mM, only Zn^2+^ significantly inhibited kiwifruit AP activity (ANOVA, *P* < .05) whereas the other metal cations tested had no significant effect. When the concentration of metal ions was increased to 10 mM, the enzyme activity was strongly inhibited by Zn^2+^ (ANOVA, *P* < .05), and inhibited to a lesser extent by Ni^2+^, Co^2+^, and Mn^2+^. At this concentration Ca^2+^ and Mg^2+^ did not have any significant effects. This suggests that the AP activity might be different from that of a previously studied protease in kiwifruit that was inhibited by calcium ions [21]. Furthermore, kiwifruit AP activity was different from that in potato, *Arabidopsis*, tomato, porcine and *E. coli* pep A as they were highly activated by Mn^2+^ and Mg^2+^ ions but were also inhibited by Zn^2+^ ions [16–18]. The kiwifruit AP activity was also different from that of grape berries which was not inhibited by EDTA, 1,10-phenanthroline, or metal ions [13].

### 3.4. Partial Purification of Kiwifruit Aminopeptidase

**Figure 6:**
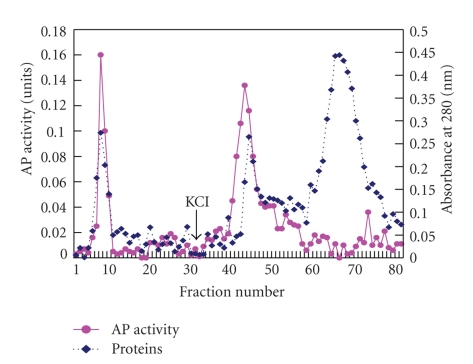
Elution profile from a DEAE cellulose column of aminopeptidase (AP) activity and protein content in a concentrated fraction of ammonium sulfate precipitation of crude extracts of *A. deliciosa* fruit. The first 30 fractions were eluted with 10 mM potassium phosphate buffer (pH 8) supplemented with 10% glycerol. The next fractions were eluted with a linear gradient of 0 to 1.0 M KCl in the same buffer. One unit of enzyme activity was defined as a change in one unit of absorbance at 410 nm per h at 37°C. Protein content was measured at 280 nm.

Two major peaks of AP activity were separated using DEAE cellulose column chromatography: the unadsorbed and adsorbed fractions (Figure 6), suggesting that there were at least two isoforms of AP activity in *A. deliciosa* fruit. In these fractions only a few low-molecular weight polypeptides were found to be present following SDS PAGE (data not shown). This might be a facile route to obtain a relatively pure food-grade aminopeptidases from kiwifruit. Further studies, using more specific substrates, could lead to some useful food-grade aminopeptidases from kiwifruit. Recombinant DNA techniques could also be applied to mass produce kiwifruit-originated APs.

## Figures and Tables

**Table 1:** Aminopeptidase activity in different parts of kiwifruit^a^.

Type of tissue	Total activity (units/g fresh weight)	Specific activity (units/mg soluble protein)
Outer pericarp	0.38 ± 0.15	0.37 ± 0.10
Inner pericarp	0.64 ± 0.19	0.52 ± 0.07
Core	2.91 ± 0.68	3.68 ± 0.96
Seed	60.15 ± 7.99	5.78 ± 0.48

^a^Aminopeptidase (AP) activity was determined in extracts of each tissue from three different fruits of *A. deliciosa*. Mean values ± standard errors are presented.
